# Smoking and drinking in relation to oral potentially malignant disorders in Puerto Rico: a case-control study

**DOI:** 10.1186/1471-2407-11-324

**Published:** 2011-07-29

**Authors:** Lin Li, Walter J Psoter, Carmen J Buxó, Augusto Elias, Lumarie Cuadrado, Douglas E Morse

**Affiliations:** 1 Department of Epidemiology & Health Promotion, New York University College of Dentistry, 250 Park Avenue South, Room 633, MC: 9479, New York, NY, 10003-1402, USA; 2 Office of the Assistant Dean of Research, School of Dental Medicine, University of Puerto Rico Medical Sciences Campus, PO Box 365067, San Juan, 00936-5067 Puerto Rico

## Abstract

**Background:**

Oral cancer incidence is high on the Island of Puerto Rico (PR), particularly among males. As part of a larger study conducted in PR, we evaluated smoking and drinking as risk factors for oral potentially malignant disorders (OPMDs).

**Methods:**

Persons diagnosed with either an OPMD (n = 86) [oral epithelial dysplasia (OED), oral hyperkeratosis/epithelial hyperplasia without OED] or a benign oral tissue condition (n = 155) were identified through PR pathology laboratories. Subjects were interviewed using a standardized, structured questionnaire that obtained information, including detailed histories of smoking and drinking. Odds ratios (ORs) for smoking and drinking in relation to having an OPMD, relative to persons with a benign oral tissue condition, were obtained using logistic regression and adjusted for age, gender, education, fruit/vegetable intake and smoking or drinking.

**Results:**

For persons with an OPMD and relative to individuals with a benign oral tissue condition, the adjusted OR for current smoking was 4.32 (95% CI: 1.99-9.38), while for former smokers, the OR_adj _was 1.47 (95% CI: 0.67-3.21), each OR_adj _relative to never smokers. With regard to drinking, no adjusted ORs approached statistical significance, and few point estimates exceeded 1.0, whether consumption was defined in terms of ever, current, level (drinks/week), or beverage type.

**Conclusions:**

In this study, conducted in Puerto Rico, current smoking was a substantial risk factor for OPMDs while former smokers had a considerably reduced risk compared to current smokers. There was little evidence suggesting that alcohol consumption was positively associated with OPMD risk.

## Background

The clinical term "oral potentially malignant disorders" (OPMDs) is used to refer to a number of clinical presentations that carry an elevated risk of oral cancer [1]. In Puerto Rico, OPMDs most frequently present as leukoplakia and erythroplakia and are often diagnosed histopathologically as oral hyperkeratosis, epithelial hyperplasia, and epithelial dysplasia (OED). Oral hyperkeratosis presents histopathologically as a thickened keratotic layer on the epithelial surface, epithelial hyperplasia is characterized by a thickening of the spinous spinosum (acanthosis) and OED presents as alterations in cellular proliferation and maturation of the oral epithelium [1-3]. Previous reports have documented the malignant potential of oral hyperkeratosis/epithelial hyperplasia and OED [4-9].

It is well-established that tobacco smoking and alcohol consumption are important independent risk factors for squamous cell carcinoma of the oral cavity and pharynx [10,11]. A number of studies in disparate areas around the world have also reported a positive association between smoking tobacco and the risk of both oral leukoplakia and OED [12]. Findings with regard to a link between alcohol consumption and OPMDs have been less consistent [12].

The purpose of the current analysis was to evaluate smoking and drinking as risk factors for OPMDs on the island of Puerto Rico where a high incidence of oral cancer is observed, particularly among males [13].

## Methods

All individuals diagnosed with an OPMD or a benign oral tissue condition were identified by reviewing pathology reports generated via pathology laboratories located in cities around Puerto Rico. The study began with one participating laboratory in February 2003; additional laboratories were added over time to a total of six in October 2007 at the conclusion of subject identification. Persons with a histopathologic diagnosis of oral hyperkeratosis, epithelial hyperplasia, or OED were categorized as having an OPMD. Seven subjects diagnosed with lichen planus were excluded from the primary analysis given the controversy over its malignant potential [14], but those subjects were included as OPMD cases in a secondary analysis. Individuals histopathologically diagnosed with one or more of the following were classified as having a benign oral tissue condition: benign adenoma, amalgam tattoo, cyst, fibroma, fibrous histocytoma, granuloma, hemangioma, incontinentia pigmenti, inflammation, benign ulceration, lipoma, mucocele, mucositis, papilloma, Sjoegren syndrome, sialandenitis, or reactive, non-epithelial hyperplasia. When a subject was diagnosed with more than one eligible oral abnormality, s/he was classified as having the more severe disease condition, e.g., if a subject was diagnosed with both a benign oral tissue condition and OED, the disease status was classified as OED.

Subjects were included in the study if they were 30+ years of age at diagnosis, had no previous history of the diagnosed oral abnormality, could speak Spanish or English, and were able to provide reliable exposure information as judged by the trained interviewers.

After obtaining permission to contact an identified individual from his/her surgeon of record, each potential subject was contacted by study personnel, first via mail, and subsequently via telephone to answer any questions regarding participation and to schedule an in-person interview appointment. Two trained interviewers, who were blinded to both study hypotheses and disease status, used a standardized, structured questionnaire that included questions on smoking, drinking, and medical/dental history as well as information on demographics and dietary habits. Each subject received $20 reimbursement for their participation time.

Smoking tobacco use was defined in terms of cigarettes, pipes, and cigars. An "ever smoker" was defined as a person who self-reported smoking at least 100 cigarettes during the course of his/her lifetime or having smoked pipes or cigars for six months or more. A "current smoker" was defined as a person who self-reported smoking within the calendar year prior to the year of diagnosis while an "ex-smoker" was defined as an ever smoker who had quit smoking for more than one calendar year prior to the diagnosis year. For instance, a subject diagnosed in 2005 who quit smoking in 2004, would be classified as a current smoker; however, the same individual would be classified as an ex-smoker if s/he quit smoking in 2003. Cigarette equivalents were calculated based on the conversion factor of 1 cigarette = 1/2 pipe = 1/4 cigar and reported in terms of average cigarette equivalents per day [15]. Pack-year equivalents of smoking was calculated based upon the average number of pack-equivalents smoked per day times the number of smoking years.

Alcoholic beverage consumption was obtained in terms of beer, wine, and hard liquor. An "ever drinker" was defined as a person who self-reported consuming at least 12 drinks of any alcoholic beverage type over his/her lifetime. A "current drinker" was defined as a person who reported drinking any type of alcoholic beverage within the calendar year prior to the diagnosis year while an "ex-drinker" was defined as an ever drinker who had discontinued all drinking for more than one calendar year prior to the diagnosis year. The average number of drinks consumed per week was calculated based upon the reported number of 12-ounce beers, 4-ounce glasses of wine, and 1.5-ounce shots of hard liquor consumed per week [15-17].

We employed standard statistical methods to evaluate differences in means and proportions. Odds ratios (ORs) and their 95% confidence intervals (95% CIs) were obtained using unconditional logistic regression [18]. Where indicated, ORs were adjusted for age (4 levels), gender, education (3 levels), daily fruit & vegetable intake (4 levels), and smoking (current vs. non-current) or drinking (4 levels). Levels of tobacco smoking (average cigarette equivalents/day, pack-year equivalents) were stratified into tertiles based upon consumption levels in the benign conditions group while the frequency of alcohol consumption was categorically defined as 0, > 0-< 7, 7-20, and > 20 drinks/wk. Crude and adjusted ORs were estimated by entering the applicable smoking and drinking terms, as factored variables, into the statistical models.

Tests of linear trend were conducted by adding the relevant ordinally-defined variable as a variate into the next lower order model and evaluating the statistical significance of that addition using the Wald statistic. An exploratory evaluation of the interaction between smoking and drinking was performed.

The study protocol was reviewed and approved by the Institutional Review Boards of the University of Puerto Rico Medical Sciences Campus and New York University.

## Results

A total of 241 subjects meeting the study eligibility criteria were included in the analysis. Table 1 presents the distribution of study participants by key variables of interest. The OPMD group (n = 86) was older than the benign group (n = 155) while the two groups were not significantly different in terms of gender, education, or fruit and vegetable intake.

**Table 1:** Distribution of demographic variables by diagnostic group

Variable	OPMDs^a^ ***n*****(%)**		Benign^b^ ***n*****(%)**
**Age**			
30-49	18 (20.9)		52 (33.5)
50-59	20 (23.3))		37 (23.9)
60-69	26 (30.2)		43 (27.7)
70+	22 (25.6)		23 (14.8)
		*P = 0.09*^c^	
Mean (SD)	60.0 (12.5)		55.6 (12.3)
		*P = 0.009*^d^	
**Gender**			
Males	40 (46.5)		58 (37.4)
Females	46 (53.5)		97 (62.6)
		*P = 0.17*^c^	
**Education**			
< 12 yrs	28 (32.6)		37 (23.9)
12 yrs/High School	15 (17.4)		29 (18.7)
> 12 yrs	43 (50.0)		89 (57.4)
		*P = 0.34*^c^	
**Fruit & vegetable servings/day**			
Mean (SD)	1.14 (0.9)		1.23 (0.9)
		*P = 0.51*^d^	
**Total**	**86**		**155**

^a ^Oral Potentially Malignant Disorders: oral hyperkeratosis, epithelial hyperplasia, and oral epithelial dysplasia

^b ^Oral Benign Tissue Conditions

^c ^Based on χ^2 ^test

^d ^Based on *t*-test

### Smoking

Among the 86 subjects diagnosed with an OPMD, 52.3% reported ever smoking cigarettes while 4.7% and 2.3% had smoked cigars or pipes, respectively. Of the 155 subjects diagnosed with a benign oral tissue condition, the corresponding percentages were 36.1%, 2.6%, and 1.9%. None of the study subjects reported using smokeless tobacco.

Table 2 presents ORs for smoking associated with having an OPMD relative to having a benign oral tissue condition. Ever versus never smoking was associated with a more than two-fold increase in the odds of having an OPMD (OR_adj _= 2.53, 95% CI: 1.33-4.80). When we stratified ever smokers in terms of ex- and current smoking, the adjusted OR was strongest for current smokers (OR_adj _= 4.32, 95% CI: 1.99-9.38) and only modestly elevated for ex-smokers (OR_adj _= 1.47, 95% CI: 0.67-3.21), linear test of trend, *P*_trend _< 0.001. When current and noncurrent (i.e., never & ex-) smokers were compared, the adjusted OR for having an OPMD relative to a benign oral condition, was 3.72 (95% CI: 1.83-7.56).

**Table 2:** Odds ratios for smoking associated with OPMDs relative to oral benign tissue conditions

Exposure	OPMDs (n)	Benign (n)	OR^a^	95% CI	OR_adj _^b^	95% CI
Never smokers	38	99	1.0	---	1.0	---
Ever smokers	48	56	2.23	1.31-3.82	2.53	1.33-4.80
						
Never smokers	38	99	1.0	---	1.0	---
Ex-smokers	17	30	1.48	0.73-2.98	1.47	0.67-3.21
Current smokers	31	26	3.11	1.64-5.90	4.32	1.99-9.38
*P_trend_*				0.001		*< 0.001*
						
Noncurrent smokers	55	129	1.0	---	1.0	---
Current smokers	31	26	2.8	1.52-5.14	3.72	1.83-7.56
						
Average cigarette equivalents/day						
0	38	99	1.0	---	1.0	---
> 0-< 20	26	27	2.51	1.30-4.83	2.83	1.35-5.93
20+	22	29	1.98	1.01-3.86	2.21	1.02-4.80
*P_trend_*			0.02			0.03
						
Pack-year equivalents of smoking						
0	38	99	1.0	---	1.0	---
> 0-< 15.5	21	29	1.89	0.96-3.70	2.26	1.06-4.83
15.5+	27	27	2.61	1.36-5.00	2.83	1.32-6.06
*P_trend_*				0.003		0.006
						
Age started smoking (Ever smokers only)						
18+	27	30	1.0	---	1.0	---
< 18	21	26	0.9	0.41-1.95	0.81	0.35-1.91

^a ^Crude OR

^b ^OR adjusted for age (four levels), gender, education (three levels), fruit & vegetable intake (four levels), and drinking (four levels)

Smoking level was defined in terms of average cigarette equivalents per day and pack-year equivalents. At each level of smoking, adjusted ORs were over twice as high for smokers relative to never smokers, and the tests of linear trend were statistically significant.

When we simultaneously included terms for both *current smoking *(current vs. noncurrent) and *average cigarette-equivalents/day *in the adjusted model, the OR for current smoking remained high (OR = 3.0, 95% CI: 1.28-7.03) while ORs for each smoking level were attenuated (i.e., adjusted ORs for average cigarette-equivalents/day declined from 1.0, 2.83, 2.21 (Table 2) to 1.0, 1.68, 1.23, *P_trend _*= 0.70). When we conducted an analogous analysis that added *current smoking *and *pack-year equivalents of smoking *to the adjusted model, the OR for current smoking was 2.95 (95% CI: 1.24-7.03), and the ORs for pack-year equivalents of smoking declined from 1.0, 2.26, 2.83 (Table 2) to 1.0, 1.48, 1.45 (*P*_trend _= 0.44).

Among ever smokers, the age at which smoking began did not differ significantly between the OPMD and benign conditions groups (OR = 0.81, 95% CI: 0.35-1.91).

### Drinking

Table 3 presents ORs for drinking in relation to having an OPMD relative to having a benign oral tissue condition. No adjusted ORs approached statistical significance, and few point estimates exceeded 1.0, whether consumption was defined in terms of ever, current, level (drinks/week), or beverage type.

**Table 3:** Odds ratios for drinking associated with OPMDs relative to oral benign tissue conditions

Exposure	OPMDs (n)	Benign (n)	OR^a^	95% CI	OR_adj_^b^	95% CI
Never drinkers	41	73	1.0	-	1.0	---
Ever drinkers	45	82	0.98	0.58-1.66	0.63	0.33-1.21
						
Never drinkers	41	73	1.0	-	1.0	---
Ex-drinkers	14	22	1.13	0.52-2.45	0.63	0.25-1.57
Current drinkers	31	60	0.92	0.52-1.64	0.63	0.32-1.26
*P*_trend_			*0.79*	*0.20*		
						
Drinks/week, all alcohol combined ^c^						
0	41	73	1.0	-	1.0	---
> 0-< 7	22	44	0.89	0.47-1.69	0.66	0.32-1.37
7-20	10	22	0.81	0.35-1.87	0.39	0.14-1.13
> 20	12	13	1.64	0.69-3.93	0.78	0.27-2.26
*P*_trend_			*0.55*	*0.32*		
Mean drinks/wk (all types combined)						
> 0	18.8 (29.9^d^)	16.2 (33.8)	*P = 0.68^e^*			
> 20	55.1 (38.0)	70.3 (58.9)	*P = 0.46^e^*			
						
Type of alcoholic beverages ^c^						
Beer, drinks/wk						
< 7	72	133	1.0	-	1.0	---
7+	14	22	1.18	0.57-2.44	0.66	0.27-1.61
Mean 12 oz beers/wk						
	9.4 (16.8)	8.1 (17.0)	*P = 0.66^e^*			
Wine, drinks/wk						
< 7	83	152	1.0	-	1.0	---
7+	3	3	1.83	0.36-9.28	1.74	0.30-10.11
Mean drinks of wine/wk						
	1.1 (2.6)	0.8(1.7)	*P = 0.44^e^*			
Hard Liquor, drinks/wk						
< 7	76	140	1.0	-	1.0	---
7+	10	15	1.23	0.53-2.87	0.62	0.23-1.65
Mean drinks of hard liquor/wk						
	8.3(22.4)	7.4(23.3)	*P = 0.84^e^*			

^a ^Crude OR

^b ^OR adjusted for age (four levels), gender, education (three levels), fruit & vegetable intake (four levels), and current smoking

^ c ^One OPMD and three benign condition subjects did not report level of drinking

^ d ^Standard deviation

^ e ^Based on *t-*test

When we compared the mean number of alcoholic drinks consumed per week for ever drinkers, persons in the OPMD group had a higher mean total consumption compared to the benign conditions group but the difference did not approach statistical significance (18.8 vs. 16.2 drinks/wk, *P *= 0.68). On the other hand, among heavy drinkers (> 20 drinks/wk), the OPMD group consumed, on average, fewer drinks/wk than the benign conditions group (55.1 vs. 70.3 drinks/wk), but the difference was not statistically significant (*P *= 0.46). After stratifying on type of alcoholic beverage, consumption was marginally higher in the OPMD group (beer: 9.4 vs. 8.1; wine: 1.1 vs. 0.8; hard liquor: 8.3 vs. 7.4); but again, not statistically significant.

### Smoking & Drinking Interaction

When we fitted an interaction term for current smoking (yes/no) and alcohol consumption (< 7, 7+drinks/week) to the adjusted model (referent category: never/ex-smokers and < 7 drinks/week), adjusted OR point estimates were 2.5 or higher for current smokers who drank either < 7 or 7+ drinks/week. For noncurrent smokers who drank 7+ drinks/week, the OR_adj _was 0.7 (Table 4).

**Table 4:** Odds ratios for smoking and drinking associated with OPMDs relative to oral benign tissue conditions

Exposure	OPMDs (n)	Benign (n)	OR^a^	95% CI	OR_adj_^b^	95% CI
Non-current smokers & < 7 drinks/wk (referent category)						
	47	107	1.0	---	1.0	---
Non-current smokers & 7+ drinks/wk						
	8	22	0.83	0.34-1.99	0.66	0.25-1.79
Current smokers & < 7 drinks/wk						
	17	13	2.98	1.34-6.62	3.58	1.53-8.37
Current smokers & 7+ drinks/wk						
	14	13	2.45	1.07-5.62	2.53	0.96-6.68

^a ^Crude OR

^b ^OR adjusted for age (four levels), gender, education (three levels), and fruit & vegetable intake (four levels)

### Additional Analyses

Point estimates for smoking and drinking did not change notably when we added variables for denture use and body mass index (BMI) to the adjusted model. In addition, adding lichen planus cases to the OPMD group had little impact on the adjusted ORs reported in Tables 2, 3 and 4. The adjusted ORs were also similar when age was entered into the model as a continuous, rather than categorical, variable.

## Discussion

Tobacco and alcohol consumption are well-recognized risk factors for cancers of the oral cavity and pharynx [12,17,19]. In Puerto Rico, it is estimated that 76% of oral and pharyngeal cancer cases among men and 52% among women are attributable to smoking and/or alcohol consumption [17]. Comparatively few epidemiologic studies, however, have investigated the role of tobacco and alcohol in relation to OPMD risk. In the current study, conducted in Puerto Rico, we evaluated smoking and drinking as risk factors for OPMDs.

### Smoking

The favored form of tobacco use can vary across geographic areas and cultures around the world. In the United States, Europe, Australia, and Japan, cigarettes, cigars, and pipes are the major types of smoking tobacco, while chewing tobacco and snuff are the most common forms of smokeless tobacco [20]. Although the prevalence of tobacco smoking is high in India, Pakistan, China, and other areas of Asia, smokeless tobacco, used either alone or as part of a concoction and in forms including betel quid, bidis, paan, naswar, or nass, is also popular [20-23].

In our study, conducted in Puerto Rico, persons smoked tobacco primarily in the form of cigarettes, with few subjects in either the OPMD or benign conditions group claiming to smoke cigars or pipes. Although smokeless tobacco use has been implicated as a risk factor for both oral leukoplakia and oral cancer on the United States mainland [24-26], in our study, no subjects with an OPMD or a benign tissue condition reported using smokeless tobacco.

In the present study and after adjusting for drinking and other potentially confounding factors, current smoking was strongly associated with OPMD risk. There was a more than four-fold increased risk among current versus never smokers; however, the risk was notably attenuated among former smokers. These findings are consistent with numerous previous studies of oral leukoplakia [21,27-32], OED [15,33], erythroplakia [34], and oral cancer [16,17,35]. Our finding of a substantially decreased OPMD risk for former smokers relative to current smokers underscores the public health benefit associated with smoking cessation.

Previous studies have reported dose- and/or duration- response relationships between smoking and the risk of oral leukoplakia, erythroplakia, and OED [15,21,34,36,37]. In the current investigation, the risk of having an OPMD increased in a monotonic fashion with increasing pack-year equivalents of smoking. The linear trend for OPMD risk in relation to average cigarette equivalents smoked per day was also statistically significant, but did not increase in a stepwise fashion, perhaps due to the relatively small OPMD sample size. Of note, our findings suggest that current smoking status is a stronger risk factor for OPMDs than reported level of smoking.

### Drinking

Alcohol consumption is an established risk factor for oral cancer [12,16,17,35]. Nevertheless, findings from investigations into the association between alcohol consumption and either oral leukoplakia or other potentially premalignant oral diagnoses have not been consistent across studies. In the current investigation, we found little evidence that overall alcohol consumption is associated with increased OPMD risk. Our results are in keeping with several earlier studies reporting inverse or null findings for total alcohol consumption [21,28,29,38,39]; but not with others that reported a positive association for either leukoplakia in general, erythroplakia, or OED [15,34,40,41].

Some studies of premalignant lesions and OED have found that risks associated with drinking are dependent upon the level of alcohol intake, with the highest risks observed for the highest level of alcohol intake [15,34,41]. After adjusting for various potential confounders; however, we found no evidence of an increased OMPD risk even among those persons who consumed > 20 drinks/wk.

Previous studies have reported that the risk of oral cancer and OPMDs can vary by beverage type, i.e., beer, wine, and hard liquor [15,16,39,42-44]. In Puerto Rico, the consumption of hard liquor, particularly when used without a mixer, has been associated with a much stronger risk of oral cancer than beer or wine [44]. In our analysis of OPMD risk, however, we found little evidence that any type of alcoholic beverage consumption was associated with an increased OPMD risk in Puerto Rico.

### Smoking and Drinking Interaction

Previous studies have reported evidence of a more than additive effect of both smoking and drinking on the risk of oral cancer and OED [12,15-17]. Our exploratory analyses revealed no evidence that alcohol consumption modified the effect of smoking in terms of OPMD risk, although this finding is based on a relatively small sample that hindered an in-depth examination of the potential interaction.

### Limitations

When interpreting our findings, it is important to consider study limitations. The relatively small sample size reflects a comparatively low rate of OPMD diagnosis in Puerto Rico [45] and limited our ability to conduct subgroup analyses related to smoking, drinking, and their interaction.

We utilized a comparison group comprised of persons diagnosed with a benign oral soft tissue condition rather than a traditional "disease-free" group. The strategy used provides an estimate of exposure patterns among persons diagnosed with asymptomatic oral lesions in Puerto Rico. It is possible, however, that one or another oral condition included in our benign conditions group is positively associated with either smoking or alcohol consumption; in that instance, the prevalence of smoking and drinking in our comparison group may exceed that of the background PR source population. Had we used a "disease-free" comparison group, ORs for smoking may have been higher than our current estimates, and ORs for drinking may have been closer to or exceeded the null value, 1.0.

Adjustment was made for a number of potential confounders, and further adjustment for denture use and BMI had little impact on the reported ORs; however, our results may reflect some uncontrolled confounding.

All exposures were measured based upon subject self-reports, and some degree of exposure misclassification may have occurred. In addition, since many OPMDs and benign oral tissue conditions are asymptomatic and may not be biopsied, we cannot rule out the possibility that undiagnosed cases are different from subjects included in the study in terms of the risk factors of interest.

## Conclusion

We compared smoking and drinking patterns between individuals diagnosed with an oral potentially malignant disorder relative to persons diagnosed with a benign oral tissue condition. Our findings suggest that current smoking is a substantial risk factor for OPMDs while former smokers have a considerably reduced risk compared to current smokers. There was little evidence suggesting that alcohol consumption was positively associated with OPMD risk in Puerto Rico.

## Competing interests

The authors declare that they have no competing interests.

## Authors' contributions

LL: conducted the statistical analysis, contributed to the interpretation of study results, and was the lead author of the manuscript. WJP: assisted in designing the study, contributed to the writing of the grant proposal that supported the research, assisted in the interpretation of study results, and contributed to the preparation of the final manuscript. CJB: served as the primary study interviewer, constructed and maintained the database, assisted in the interpretation of study results, and contributed to the writing of the final manuscript. AE: assisted in designing the study, in overseeing study activities in Puerto Rico, and contributed to writing of the final manuscript. LC: generated all study documents, coordinated day-to-day study activities in Puerto Rico, and maintained the overall study database. DEM: as Principal Investigator, designed the study, wrote the grant proposal that supported the research, assisted with the statistical analysis, and contributed to interpretation of study results and preparation of the final manuscript.

All authors read and approved the final manuscript.

## Pre-publication history

The pre-publication history for this paper can be accessed here:

http://www.biomedcentral.com/1471-2407/11/324/prepub
